# Relationships between obesity markers and bone parameters in community-dwelling older adults

**DOI:** 10.1007/s40520-023-02673-8

**Published:** 2024-02-29

**Authors:** L. Lemoine, F. Buckinx, A. Aidoud, V. Leroy, B. Fougère, M. Aubertin-Leheudre

**Affiliations:** 1 Division of Geriatric Medicine, Tours University Medical Centre, Tours, France; 2 EA4245 T2i, Université de Tours, Tours, France; 3 EA 7505 Education, Ethics, Health, Tours University, Tours, France; 4 Département des Sciences de l’activité Physique, Faculté des Sciences, Groupe de recherche en Activité Physique Adaptée (GRAPA), Université du Québec À Montréal, Montreal, QC Canada; 5 Centre de recherche de l’Institut, Université de Gériatrie de Montréal, Montreal, QC Canada; 6 CHRU Tours – Service de Médecine Aigue Gériatrique, Hôpital Bretonneau, 2 Boulevard Tonnellé, 37044 Tours Cedex 9, France

**Keywords:** Fat mass, Bone density, Bone architecture, Aging, Obesity

## Abstract

**Background:**

Osteoporosis is an age-related condition that can lead to fragility fractures and other serious consequences. The literature data on the impact of obesity on bone health are contradictory. The main reasons for this discrepancy could be the imperfect nature of the body mass index (BMI) as a marker of obesity, the metabolic status (inflammation and metabolically healthy obesity), and/or heterogeneity in bone variables and architecture or sex.

**Aims:**

To examine the relationship between bone variables and three validated obesity criteria.

**Methods:**

In this cross-sectional study, participants were classified as obese according to their BMI, waist circumference (WC), and fat mass (FM). Bone variables and architecture were assessed using dual-energy X-ray absorptiometry and peripheral quantitative computed tomography, respectively.

**Results:**

One hundred sixty-eight adults aged 55 or over (men: 68%) were included. 48 (28%) participants were obese according to the BMI, with 108 (64%) according to the FM, and 146 (87%) according to the WC. Bone variables were positively correlated with WC and BMI (Pearson’s *r* = 0.2–0.42). In men only, the obesity measures were negatively correlated with cortical bone density (Pearson’s *r* = − 0.32 to − 0.19) and positively correlated with cortical bone area (Pearson’s *r* = 0.22–0.39).

**Conclusion:**

Our findings indicate that independent of sex and obesity criteria, when significant, being obese seems to lead to higher bone parameters than being non-obese, except for cortical bone density. Thus, in the obese population, assessing cortical density might help the physician to identify bone alteration. Further researches are needed to confirm our findings.

**Supplementary Information:**

The online version contains supplementary material available at 10.1007/s40520-023-02673-8.

## Introduction

Osteoporosis is an age-related disease characterized by low bone mass and micro-architectural deterioration of the bone tissue, which leads to bone fragility and fractures. The estimated prevalence of osteoporosis are 15% in men and 30% in women over the age of 50 and 46% and 77% in men and women over the age of 80 [1]. In view of population aging worldwide, osteoporosis is considered to be a major public health issue. Indeed, the number of fractures (including hip fractures) is expected to rise by 50% between 2000 and 2050 [2, 3]. Osteoporotic fracture is known to increase the risk of death [4], the loss of physical autonomy, and the likelihood of hospital admission. In Europe, the estimated economic burden of osteoporotic fracture is 37 billion euros per year [5].

Low bone mineral density (BMD, typically estimated with dual-energy X-ray absorptiometry (DXA)) is directly and linearly associated with fracture risk [6]*.* However, low BMD does not fully account for the increase in the incidence of hip fracture with age [7]. Bone strength and bone architecture are biomarkers of fracture risk. Furthermore, a number of factors not associated with age (e.g. biological sex) or weakly associated with age (e.g. nutrition (vitamin D/calcium deficiencies) and body composition (fat and muscle masses)) are linked to the osteoporosis fracture risk [8].

However, the literature data on osteoporosis in obese patients are contradictory. For example, it has been reported that obesity protects against a decrease in hip and spine BMD (i.e. osteoporosis status) [9] and fracture [10]. Furthermore, a study in post-menopausal women showed that a higher body mass index (BMI) was associated with a higher BMD but lower indices of bone strength [11, 12]. One explanation for the higher BMD and fewer osteoporotic fractures in obese people relates to greater skeletal loading and tissue padding [13]. Nevertheless, other studies came to the opposite conclusion; for example, it has been reported that obesity can increase the fracture risk as a result of (i) greater impact forces during a fall [12] and (ii) the secretion of pro-inflammatory cytokines that harm bone tissue [14].

The discrepancies in the literature data might be due to phenotypic differences between obese study populations, i.e. differences in fat distribution (visceral vs. subcutaneous) and the prevalence of metabolic complications. Different types of fat (e.g. subcutaneous vs. visceral, or gynoïd/appendicular vs. androïd) have different health consequences, as metabolic syndrome is especially linked to visceral or android fat. Given that and that aging can cause a change in body composition without a change in body weight [15], the BMI might not be a perfect guide to body fat content [16]. Accordingly, waist circumference (WC, which reflects android obesity) is associated with an elevated risk of hip fracture [17]. Furthermore, fat mass (FM) is considered to be a better clinical marker than BMI of obesity in older adults [18]. Similar results have been reported for a high FM and fracture risk [19]. Around 20% of obese people are metabolically healthy [20], and some researchers use the term “metabolically healthy obesity” [21]. In contrast, around 10% of obese people have a low muscle mass and low strength; this “sarcopenic-obesity” is known to have an impact on bone health and is associated with an elevated fracture risk in older men [22] and low BMD [23].

Another possible explanation for the discrepancies in the literature data is that BMD might not be the best proxy marker of bone fragility. Indeed, a study of a cohort of obese women (according to the BMI) with a low trauma fracture found that only 12% had osteoporosis according to DXA [24]. Changes in bone microarchitecture (measured by peripheral quantitative computed tomography (pQCT)) were found to predict fracture more accurately [25]. Thus, evaluating bone architecture with pQCT appears to be clinically relevant.

Lastly, biological sex and hormonal status in bone variables need to be considered. The bone turnover induced by menopause means that women have a higher risk of osteoporosis and fracture than men [26]—although the gap narrows with age [27]. Thus, bone mass is higher in men than in women but the BMD is similar in the two sexes [28]. The estimated fracture risk as a function of BMD is the same in men and women [27, 29]. Furthermore, cortical BMD and bone strength fall more quickly with age in women than in men [30, 31].

Despite osteoporosis’s clear impact on mortality, morbidity, and healthcare costs, the disease is still underdiagnosed and undertreated [32]. The accurate identification of individuals at risk—and especially obese older adults—should therefore be a priority. Thus, the objective of the present study was to assess the relationship between body composition and bone variables in community-dwelling older adults. Our hypothesis was that obesity, measured differently than with BMI, could negatively impact bone architecture.

## Materials and methods

We performed an a-posteriori cross-sectional study. All procedures were approved by the research ethics board at the Université du Québec à Montréal (Montréal, Québec, Canada). All participants provided their written consent after having received information about the study.

### Study population

The study sample was selected from 2 previous studies, conducted in our laboratory setting, which recruited community-dwelling older adults (only men in one study [33] and men and women in the other [34]) between 2016 and 2018. Among this number, 168 participants with all bone and obesity outcomes at baseline were included in this secondary analysis. Participants were recruited via adverts displayed in the Montréal area.

The main inclusion criteria were as follows [33, 34]: age 60 or over; not physically active (< 120 min/week of structured exercise); stable body weight (± 2 kg) over the previous 12 months; absence of menses for at least the previous 12 months; non-smoker status; low alcohol consumption (≤ 2 alcoholic drinks/day); community-dwelling. Exclusion criteria included [33, 34]: non-stable chronic disease (neurological, cardiovascular or cognitive disorders) and BMI < 19 kg/m^2^. These criteria were chosen to minimize the heterogeneity of the study population and to mitigate the potential impact of pre-existing medical conditions.

### Criteria for obesity

Obesity was defined with regard to three validated clinical markers: BMI (the clinical gold standard) ≥ 30 kg/m^2^ [35]), total FM (measured with DXA and expressed as a percentage of body fat/bodyweight) > 28% for men and > 40% for women [18], and WC (> 94 cm for men and > 80 cm for women [36]).

Metabolic status was assessed with regard to the systolic and diastolic blood pressure and standard laboratory variables, including the serum levels of triglycerides (mmol/l), high-density lipoprotein (HDL) cholesterol (mmol/l) and fasting glucose (mmol/l). Metabolic syndrome was defined according to the International Diabetes Foundation criteria [36] or the National Cholesterol Education Program (NCEP) criteria (less strict criteria) [37].

### Data collection

The data were collected by highly trained clinical assessors at the university’s Department of Exercise Science. Body weight (kg) and height (m) were determined in the fasting state, using an electronic scale (GFK 660a, Adam Equipment Inc, Oxford, UK) and a stadiometer (Seca©, Hamburg, Germany). Thereafter, the BMI (body mass (kg)/height (m^2^)) was estimated. With the participant standing upright, waist circumference was measured to the nearest 0.5 cm using a flexible but non-stretchable measuring tape.

Total FM (%), total, hip and spine BMD (g/cm^3^), arm, leg and total bone mineral content (g), and appendicular lean mass (ALM) were quantified with DXA (GE Medical Systems, Madison, WI, USA).

Osteoporosis status (bone fragility) was determined using T-scores at total, hip and spine sites. A T-score between + 1 and − 1 corresponded to normal BMD, a T-score between − 1 and − 2.5 corresponded to osteopenia, and a T-score of − 2.5 or less at the hip or spine site corresponded to osteoporosis. For the evaluation of body composition (in the supine position), participants were asked to fast before the examination and to remove all jewellery. Low muscle mass was defined according with regard to the ALM (measured using DXA) and the European Working Group on Sarcopenia in Older People sarcopenia cut-off (ALM < 20 kg for men and < 15 kg for women) [38].

Using pQCT data (Stratec XCT3000; STRATEC Medizintechnik GmbH) for the right femur (one-third of the distance between the lateral epicondyle and the greater trochanter), we obtained thresholds for BMD, cortical bone density (mg/cm^3^), cortical bone area (mm^2^), and two other biomechanical indexes: the strength strain index (SSI, mm^3^), whereas the polar second moment of area (IPo, mm^4^) reflects torsion and flexion [39]. IPo refers to the fourth power of length (mm4) in the context of an area moment of inertia. It takes into account both the quantity and distribution of bone mass and quantifies the resistance of the bone to bending and torsional forces. The higher the value in mm4, the greater the bone's resistance to bending and torsional loading. All scans were performed by operators trained in how to acquire pQCT data in accordance with the guidelines published by the company Bone Diagnostic (Fort Atkinson, WI, USA). The results were produced automatically, using ImageJ analysis (version 1.3.11) [39].

### Statistical analyses

Baseline characteristics were summarized using descriptive statistics. All continuous variables were expressed as the mean ± standard deviation (SD) and categorical variables in percent. The normality of distribution was checked in the Kolmogorov–Smirnov test (*p* > 0.05), and the skewness and kurtosis (± 2) distributions were calculated for all variables for the men and for the women separately. Cortical density among the men was the only non-normally distributed variable.

Based on the normality of distribution, Pearson’s or Spearman’s two-tailed correlation test was applied to determine the relationship between adipose markers (BMI, FM, or WC) and bone variables. To interpret the coefficients of correlation, we used the following validated classification [40]: weak: *r* < 0.5 vs. moderate: *r* = 0.5–0.7 vs. strong: *r* > 0.7. For significant correlations, we also performed a correlation by sex [41].

We performed *t*-test comparisons to assess differences in adipose and bone characteristics based on obesity status (obese vs. non-obese) across the three obesity criteria (WC; FM and BMI), and based on metabolic syndrome status (MS vs. No-MS; IDF criteria). As recommended, logistic regression was also used to evaluate whether any of the obesity criteria (WC, FM, and BMI) could predict bone fragility.

All statistical analyses were performed using SPSS software (version 27.0, IBM Corp., Armonk, NY, USA). The threshold for statistical significance was set to *p* < 0.05.

## Results

**Fig. 1:**
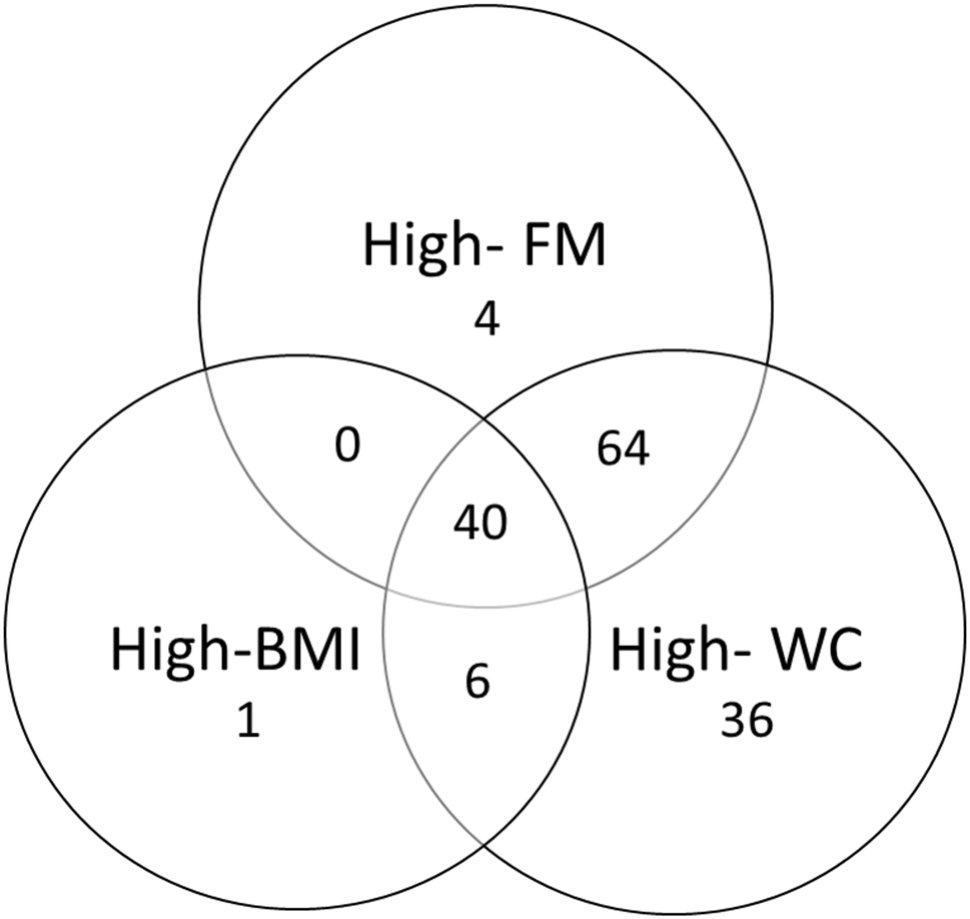
Distribution of obesity in the study population according to the different measures (*n* = 151). BMI: body mass index; WC: waist circumference; FM: fat mass

A total of 168 participants were included in the study (Table 1). The mean age was 68 ± 7, the majority of the participants were men (68%), and 66 participants (39%) were considered to have bone fragility (osteoporosis or osteopenia). Using the thresholds defined above, the proportion of the study participants considered to be “obese” was 28% (*n* = 48) with the BMI threshold, 64% (*n* = 108) with the FM threshold, and 87% (*n* = 146) with the WC threshold. Notably, 24% of the participants (*n* = 40, including 22 men) were classified as “obese” according to all three indices, and 10% (*n* = 17, including 16 men) were not classified as “obese” according to any of the indices (Fig. 1). As shown in supplemental tables (Table S1 and Table S2), we observed, in both sexes, that obese participants had significantly higher BMI, WC and/or fat mass content than non-obese participants. It is noteworthy that a comparison between obese and non-obese women based on WC was not performed as only 1 woman was classified as non-obese using these criteria (obese: *n* = 52 vs. non-obese: *n* = 1).”

**Table 1:** Main characteristics of the study participants (*n* = 168)

Variables	All participants (*n* = 168)	Men (*n* = 114)	Women (*n* = 54)	*p*-value
*General characteristics*				
Age (years)	68 ± 7.48	68 ± 5.8	67 ± 4.1	NS
WC (cm)	102 ± 12	103 ± 11	99 ± 13	0.043
Prevalence of obesity, according to the WC (n)	146 (87%)	93 (82%)	53 (98%)	0.002
BMI (kg/m^2^)	28 ± 4.7	28 ± 3.9	29 ± 6	NS
Prevalence of obesity, according to the BMI (n)	48 (28%)	29 (25.4%)	19 (35%)	NS
Systolic blood pressure (mmHg)	127 ± 14	128 ± 13	125 ± 16	NS
Blood glucose (mmol/l)	5.74 ± 1.04	5.9 ± 1.14	5.41 ± 0.7	< 0.001
HDL-cholesterol	1.44 ± 0.31	1.35 ± 0.26	1.61 ± 0.34	< 0.001
Triglycerides (mmol/l)	1.34 ± 0.7	1.31 ± 0.65	1.40 ± 0.73	NS
Metabolic syndrome	65 (39%)	44 (39%)	21 (39%)	NS
*Body composition (DXA)*				
Total FM (%)	34 ± 8.7	30.7 ± 4	42 ± 7	< 0.001
Prevalence of obesity, according to the FM (n)	108 (64%)	77 (67.5%)	31 (60%)	NS
Total BMD (g/cm^2^)	1.203 ± 0.11	1.238 ± 0.09	1.134 ± 0.12	< 0.001
Spine BMD (g/cm^2^)	1.198 ± 0.21	1.249 ± 0.18	1.094 ± 0.22	< 0.001
Hip BMD (g/cm^2^)	1.019 ± 0.15	1.056 ± 0.13	0.947 ± 0.15	< 0.001
Total T-score	0.085 ± 1.22	0.184 ± 1.19	− 0.155 ± 1.26	NS
Spine T-score	− 0.008 ± 1.64	0.254 ± 1.49	− 0.637 ± 1.84	0.002
Hip T-score	− 0.374 ± 1.07	− 0.281 ± 1.03	− 0.595 ± 1.15	NS
Arm BMC (g)	0.380 ± 1.03	0.434 ± 0.07	0.267 ± 0.06	< 0.001
Leg BMC (g)	1.128 ± 0.20	1.227 ± 0.15	0.925 ± 0.14	< 0.001
Prevalence of osteopenia/osteoporosis (n)	66 (39%)	44 (41%)	22 (43%)	NS
Prevalence of osteoporosis (n)	21 (12%)	9 (6%)	12 (22%)	0.012
Appendicular lean mass (kg)	23 ± 4.7	26 ± 3	18 ± 2	< 0.001
Prevalence of low muscle mass (n)	30 (18%)	24 (21%)	6 (11%)	NS
*Bone variables (pQCT)*				
Cortical bone density (mg/cm^3^)	1095 ± 33	1100 ± 31.7	1085 ± 34	0.002
Cortical bone area (mm^2^)	398 ± 60	425 ± 49	342 ± 39	< 0.001
Total bone density (mg/cm^3^)	711 ± 92	713 ± 88	705 ± 100	NS
Total bone area (mm^2^)	648 ± 106	688 ± 89	558 ± 81	< 0.001
SSI (mm^3^)	3052 ± 681	3398 ± 523	2367 ± 365	< 0.001
IPo (mm^4^)	57 862 ± 16 355	65 405 ± 14 112	43,056 ± 8412	< 0.001

Data are presented as the mean ± SD or n (%)

*WC* waist circumference; *BMI* bone mass index; *BMD* bone mass density; *BMC* bone mineral content; *FM* fat mass; *SSI* strength strain index; *IPo* polar second moment of area

*For comparisons of men and women, *p*-values were calculated in a chi-squared test or a t-test

Although 40% (*n* = 67, including 40 men) of the participants were classified as non-obese according to the BMI, they presented a high FM (36.2 ± 5.2%). Furthermore, 18% (*n* = 30) of the participants were classified as having sarcopenia.

One participant was classified as “sarcopenic obese” according to the BMI threshold, 5 (3%) according to the WC threshold and 6 (3.5%) according to the FM threshold. Based on the IDF criteria, 39% of the participants presented metabolic syndrome (with no difference in the proportions of men vs. women). The fasting glucose level was higher in men than women (5.90 vs. 5.41 mmol/l, respectively; *p* < 0.001), and the serum HDL-cholesterol was lower in men than women (1.35 vs. 1.61 mmol/l, respectively; *p* < 0.001). When using the NCEP cut-off, 22% of the women (*n* = 12) and 27% of the men (*n* = 34) had metabolic syndrome (MS; data not shown). However, there were no significant differences in bone parameters between women with metabolic syndrome (MS: *n* = 21) and those without metabolic syndrome (No-MS: *n* = 32). Similar results were found in men (No-MS: *n* = 58 vs. MS: *n* = 41) except for total bone mineral density (BMD), which was significantly higher among men with metabolic syndrome (*p* < 0.05; data not shown).

### Bone parameters comparison between obese and non-obese groups according to obesity criteria

#### Men (see supplemental Table S1)

In men, those classified obese based on BMI (obese: *n* = 29 vs. non-obese: *n* = 85) exhibited significantly higher bone parameters for cortical area (CoA) and torsion indexes (SSI & IPo) than those non-obese. Similarly, men classified as obese based on WC (obese: *n* = 93 v. non-obese: *n* = 21) had significantly higher spine and total BMD as well as cortical area (CoA) and density (CoD) than non-obese men. When obesity is categorized using FM (obese: *n* = 77 vs. non-obese: *n* = 37), obese men displayed significantly lower cortical density (CoD) than non-obese men. No other difference was observed in men.

#### Women (see supplemental Table S2)

In women, those classified as obese based on BMI (obese: *n* = 19 vs. non-obese: *n* = 35) exhibited significantly higher bone parameters than non-obese women except for cortical density (CoD) and area (CoA), and total bone density. As mentioned previously, it was not possible to compare obese and non-obese women based on WC as only 1 woman was classified as non-obese using these criteria (obese: *n* = 52 vs. non-obese: *n* = 1). Finally, women classified as obese based on FM (obese: *n* = 31 vs. non-obese: *n* = 23) displayed significantly higher bone parameters, except for cortical density (CoD) and area (CoA), as well as total bone density and spine BMD.”

### The relationship between body composition and bone parameters

#### Men (Table 2)

**Table 2:** Correlations between obesity factors, bone variables, and body composition in men (*n* = 114)

Variable	BMI	WC	FM
*Anthropometric data*			
BMI (kg/m^2^)	1	0.785 (0.703;0.847) ***	0.792 (0.712;0.852) *
WC (cm)	0.785 (0.703;0.847) ***	1	0.738 (0.642; 0.812) *
*Body composition (DXA)*			
Total FM	0.792 (0.712; 0.852) ***	0.738 (0.642; 0.812) ***	1
Total BMD (g/cm^2^)	0.266 (0.082.; 0.432) **	0.203 (0.015; 0.377) *	− 0.118 (− 0.072; 0.299)
Spine BMD (g/cm^2^)	0.045 (− 0.145.; 0.232)	0.051 (− 0.139; 0.238)	− 0.027 (− 0.215; 0.163)
Hip BMD (g/cm^2^)	0.144 (− 0.046; 0.324)	0.036 (− 0.139; 0.238)	0.064 (− 0.126; 0.250)
Arm BMC (g)	0.004 (− 0.181; 0.190)	− 0.071 (− 0.253; 0.116)	− 0.067(− 0.249; 0.120)
Leg BMC (g)	0.165 (− 0.021; 0.340)	0.076 (− 0.111; 0.258)	0.021 (− 0.165; 0.205)
Spine T score	0.054 (− 0.0149; 0.251)	0.081 (− 0.121; 0.277)	− 0.017 (− 0.216; 0.184)
Hip T score	0.114 (− 0.089; 0.307)	0.055 (− 0.147; 0.253)	0.030 (− 0.172; 0.229)
Total T score	0.262 (0.066; 0.439) **	0.247 (0.050; 0.426) *	0.097 (− 0.104; 0.291)
Appendicular lean mass (kg)	0.333 (0.157; 0.489)***	0.203 (0.018; 0.375) *	− 0.040 (− 0.224; 0.147)
*Bone variables (pQCT)*			
Cortical bone density (mg/cm^3^)*	− 0.253 (− 0.427; − 0.060)**^§^	− 0.322 (− 0.487; − 0.136) ***^§^	− 0.194 (− 0.375; − 0.020) *^§^
Cortical bone area (mm^2^)	0,389 (0,209; 0,544)***	0,220 (0,025;0,399) *	0,218 (0,023; 0,398) *
Total bone density (mg/cm^3^)	0.083 (− 0,108; 0,269)	− 0.019 (− 0.208; 0.172)	0.112 (− 0.079; 0.212)
Total bone area (mm^2^)	0.205 (0,011; 0,384)*	0.158 (− 0.038; 0.342)	0.019 (− 0.176; 0.212)
SSI (mm^3^)	0.231 (0.043; 0.404)*	0.105 (− 0.087; 0.289)	0.034 (− 0.157; 0.222)
IPo (mm^4^)	0.277 (0.091; 0.444)**	0.177 (− 0.015; 0.355)	0.093 (− 0.100;0.278)

Correlations were conducted using Pearson's correlation test, and results were presented with confidence intervals. For correlations indicated by the § symbol, Spearman's correlation test was employed, depending on the normality of the data distribution

*BMI* body mass index; *WC* waist circumference; *FM* fat mass, *BMD* bone mass density; *BMC* bone mineral content; *SSI* strength strain index; *IPo* polar second moment of area

*p < 0.05, **p < 0.01, ***p < 0.001

The correlations between BMI and total BMD, total T-score, cortical bone density or cortical bone were weak (*r* < 0.3) but statistically significant (*p* < 0.05). Likewise, the correlations between WC and bone variables were weak but significant. All adiposity measures (BMI, WC and FM) were negatively correlated with cortical bone density (BMI: *r* = − 0.25, *p* = 0.009; WC: *r* = − 0.32, *p* = 0.001 and FM: *r* = − 0.194, *p* = 0.046; respectively) and positively correlated with cortical bone area (*r* = 0.39, *p* < 0.001; *r* = 0.22, *p* = 0.028 and *r* = 0.22, *p* = 0.029; respectively). The biomechanical indexes were only moderately correlated with BMI (SSI: *r* = 0.23, *p* = 0.0016; dwIPo: *r* = 0.257, *p* = 0.008; IPo: *r* = 0.28; *p* = 0.004). Lastly, the ALM was correlated positively with BMI and WC (*r* = 0.33, *p* < 0.001 and *r* = 0.20, *p* = 0.032, respectively) but not with FM.

#### Women (Table 3)

**Table 3:** Correlations between obesity factors, bone variables, and body composition in women (*n* = 54)

Variable	BMI	WC	FM
*Anthropometric data*			
BMI (kg/m^2^)	1	0.894 (0.823;0.938) ***	0.820 (0.708;0.892) ***
WC (cm)	0.894 (0.823; 0.938) ***	1	0.729 (0.571; 0.835) ***
*Body composition (DXA)*			
Total FM	0.820 (0.708; 0.892) ***	0.729 (0.571; 0.835) ***	1
Total BMD (g/cm^2^)	0.402 (0.150; 0.604) **	0.421 (0.170; 0.621) **	0.224 (− 0.047; 0.464)
Spine BMD (g/cm^2^)	0.323 (0.060; 0.543) *	0.285 (0.016; 0.516) *	0.214 (− 0.057; 0.456)
Hip BMD (g/cm^2^)	0.337 (0.076; 0.554) *	0.343 (0.080; 0.561) *	0.245 (− 0.025; − 0.481)
Arm BMC (g)	− 0.010 (− 0.282; 0.264)	0.086 (− 0.195; 0.353)	− 0.064 (− 0.331; 0.213)
Leg BMC (g)	0.168 (− 0.104; 0.417)	0.229 (− 0.044; 0.470)	0.113 (− 0.159; 0.370)
Spine T score	0.277 (− 0.037; 0.542)	0.242 (− 0.076; 0.514)	0.212 (− 0.107; 0.491)
Hip T score	0.222 (− 0.097; 0.499)	0.238 (− 0.079; 0.512)	0.211 (− 0.107; 0.490)
Total T score	0.286 (− 0.028; 0.549)	0.306 (− 0.006; 0.564)	0.201 (− 0.118; 0.482)
Appendicular lean mass (kg)	0.404 (0.150; 0.608) *	0.444 (0.195; 0.639) ***	0.127 (− 0.148; 0.384)
*Bone variables (pQCT)*			
Cortical bone density (mg/cm^3^)	− 0.288 (− 0.516; − 0.022) *	− 0.233 (− 0.473; − 0.040)	− 0.220 (− 0.461; 0.050)
Cortical bone area (mm^2^)	0,103 (− 0,190; 0,379)	0,139 (− 0.158;0.412)	0,071 (− 0.220; 0.351)
Total bone density (mg/cm^3^)	− 0.171 (− 0.420; 0.101)	− 0.161 (− 0.414; 0.114)	− 0.120 (− 0.376; 0.153)
Total bone area (mm^3^)	0.367 (0,086; 0,594)*	0.434 (0.161; 0.645) **	0.246 (− 0.048; 0.501)
SSI (mm^3^)	0.188 (− 0.084; 0.434)	0.271 (0.001; 0.504) *	0.149 (− 0.124; 0.401)
IPo (mm^4^)	0.340 (0.079; 0.557)*	0.373 (0.114; 0.584) **	0.234 (− 0.298;0.237)

Correlations were conducted using Pearson's correlation test, and results were presented with confidence intervals

*BMI* body mass index; *WC* waist circumference; *FM* fat mass, *BMD* bone mass density; *BMC* bone mineral content; *SSI* strength strain index; *IPo* polar second moment of area

*p < 0.05, **p < 0.01, ***p < 0.001

BMI and WC were significantly and positively correlated with total BMD, spine BMD, hip BMD and appendicular LM (for BMI: *r* = 0.40, *p* = 0.003; *r* = 0.32, *p* = 0.017; *r* = 0.34, *p* = 0.013 and *r* = 0.40, *p* = 0.0003; respectively; for WC: *r* = 0.42, *p* = 0.012; *r* = 0.29, *p* = 0.039; *r* = 0.34, *p* = 0.012 and *r* = 0.44, *p* = 0.001; respectively).

BMI was negatively correlated with cortical density (*r* = − 0.29, *p* = 0.035) but positively correlated with total bone area (*r* = 0.37, *p* = 0.012). WC gave similar results, but the correlation was not significant for cortical density (cortical density *r* = − 0.23, *p* = 0.09; total bone area: *r* = 0.158; *p* = 0.003).

The biomechanical indexes were moderately correlated with WC (SSI: *r* = 0.27, *p* = 0.05; dwIPo: *r* = 0.32, *p* = 0.02; IPo: *r* = 0.37; *p* = 0.006). Furthermore, IPo was correlated with BMI (*r* = 0.34, *p* = 0.012). The correlations between FM and the bone variables were similar to those seen for the two other adiposity measures but none were statistically significant.

Correlations between BMI and cortical area showed a significant difference according to sex (*Z* = 1.8, *p* = 0.035). Correlations between WC and total area showed also a significant difference according to sex (*Z* = 1.8, *p* = 0.035). No other significant correlation, according to sex was observed (data not shown).

As recommended, we explored the importance of obesity criteria to predict bone fragility in men and women using a logistic regression analysis. We did not observe any obesity criteria that significantly predicted bone fragility (see Table 4). Thus, the level of prediction of these obesity criteria on bone fragility should be considered as low or moderate.

**Table 4:** Logistic regression to determine the predictor of bone fragility based on obesity criteria

Dependent variables	All participants (*n* = 168)	Men (*n* = 114)	Women (*n* = 54)
*Bone fragility (defined as T-score* < − *1)*			
BMI	0.924 (0.439; 1.947)	0.989 (0.365; 2.679)	0.810 (0.191; 3.438)
WC	0.669(0.244; 1.833)	0.525 (0.487; 3.697)	–
FM	0.878(0.424; 1.818)	1.342 (0.165; 1.671)	0.535 (0.135; 2.118)

Results are presented as odd ratio and confidence interval. BMI, WC and FM was considered as categorical variables. Logistic regression was not possible for waist circumference in women because the number of non-obese women was too small

*BMI* body mass index; *WC* waist circumference; *FM* fat mass

## Discussion

The present study showed that obesity was associated with altered microarchitecture in men population. Other results of our study are also worth mentioning.

Firstly, the prevalence of obesity in our study population varied markedly as a function of the chosen criteria. We expected FM-obesity to be more prevalent than BMI-obesity, given the known age-related changes in body composition [42]. In a population of older adults, it was reported that the prevalence of obesity (calculated according to the FM) ranged from 40 to 50%, depending on the sex [43]. In addition, the Institut National de Santé Publique du Québec reported that abdominal obesity concerns about 40% of the adult population and BMI-based obesity concerns only 25%. Furthermore, more than a third of the participants in the present study had a high FM but were not BMI-obese. Our findings are in line with the literature data but were even more pronounced in other studies [44].

As hypothesized, a high BMI was associated with greater BMD. This association was also observed with WC, whereas a high FM was not directly associated with BMD. In a study of postmenopausal women, Sharma et al. reported that visceral FM was negatively correlated with BMD (*r* = − 0.368, *p* = 0.017) after controlling for BMI [45], which could explain different results from our study. Scott et al. showed that high-FM participants had a higher osteoporosis risk than all other participants, regardless of their BMI-obesity status [43]. In our study, MS in men was associated with greater BMD, which was consistent with a previous meta-analysis [46]. A recent study showed that the association tended to be attenuated after adjustment for BMI, suggesting that this association is mediated by BMI [47]. Also, the parameter that could have a negative effect was blood glucose [46], making it the potentially relevant metabolic marker of the negative effect on bone.

In men, all three obesity criteria were moderately correlated with cortical bone density and cortical bone area. These results contrasted in part with those observed in women, with a significant difference concerning bone area. Thus, in men, obesity might alter the cortical microarchitecture. This finding is important because previous research showed that cortical BMD is low in the obese population (defined using BMI) [48] and is related to the severity and/or number of fractures [25].

Furthermore, it is well known that bone parameters estimated using pQCT are sometimes more relevant than bone parameters (density) estimated using DXA to detect bone alteration but also more strongly related to osteoporosis and fracture risks [24, 49].

In addition, our results are congruent with a recent study that showed that lower limb fracture was associated with obesity according to different criteria, depending on sex. In men, BMI-obesity was associated with lower limb fracture but it was WC-obesity in women [50]. Adiposity markers and microarchitecture features tended to be weakly negatively correlated in men population of our study. It is not clear from the literature whether the effect of obesity on bone depends on sex. Some studies did not find a sex difference [51], whereas others showed that the correlation between FM and BMD was stronger in women than in men [52, 53]. Conversely, a recent study showed that men with sarcopenic obesity have worse bone variables than women [54].

There are several explanations for the potentially harmful impact of obesity on microarchitecture in men. First, even if the prevalence of metabolic syndrome was similar in men and women, fasting glycemia was higher in men—suggesting greater insulin resistance. It is known that glucose metabolism and bone metabolism can influence each other [55]. Recently, a study of adolescents showed that insulin resistance was negatively correlated with BMD [56]. Moreover, obesity is known to induce systemic inflammation through greater secretion of pro-inflammatory cytokines like tumour necrosis factor-alpha and interleukin-6 [57]. This inflammation appears to be particularly related to metabolic syndrome and insulin sensitivity [58, 59]. The chronic low-grade inflammation observed in obesity is also observed in aging as the so-called “inflammaging”; the latter has a role in the development of age-related diseases, even though this has not been formally demonstrated for osteoporosis [60]. Nevertheless, proinflammatory cytokines stimulate bone resorption and lead to bone loss in the long term [14]. Osteosarcopenic obese adults and non-obese adults with sarcopenia display elevated levels of inflammatory markers [61, 62]. As our participants were in good health (due to our inclusion conditions) since between 60 and 75% (depending on the criterion) did not present metabolic syndrome and more than 80% had a normal muscle mass. As our participants are voluntary they exhibited better health (majority are non-sarcopenic and the prevalence of metabolic syndrome was similar to the general population) than expected in the obese population which can lead to reduced chronic low-grade inflammation. This difference could potentially account for the absence of expected negative correlations across all parameters.

Overall, our findings in addition to the previous literature underscore the importance of not considering obesity only as a status, but also to integrate the metabolic disorders related to, in the fat-bone paradox. Even though some obese people appear to be protected from osteoporotic bone fractures, many others are not. Moreover, obese people suffering from osteoporotic fractures are less likely to receive specific treatment [63], and so this population needs to be targeted more precisely. We did not observe a detrimental effect on bone health based on any specific measure of obesity, contrary to our initial suspicion.

However, the present study had some limitations. Firstly, the cross-sectional nature of the study prevented us from identifying causal relationships. Secondly, the large number of bivariable comparisons performed might have increased the risk of false positives. It's critical to note that while some variables may appear to have opposite trends, the correlation values are often < 0.50, indicating a weak relationship.

Thirdly, the a-posteriori design means that we did not have data on certain confounding factors (such as prior fractures, age at menopause), obesity history, and past physical activity level. Furthermore, the study lacked sufficient statistical power to facilitate a meaningful comparison between sexes; thus, the comparisons presented in Table 1 should be considered as indicative rather than conclusive. It is noteworthy that there is no consensus on the FM cut-off for obesity, which leads to very varied prevalence in the literature [64]. The present study also had a number of strengths. Firstly, we used gold-standard tools (pQCT and DXA) to measure bone density and architecture. Secondly, the present study was one of the first to have compared three clinically validated obesity criteria and, to the best of our knowledge, the first to have examined the relationship between obesity and the pQCT microarchitecture in male adults [65].

## Conclusion

Overall, our results indicate that, regardless of sex and obesity criteria, when a significant difference was found between obese and non-obese, obesity appeared to be associated with higher bone parameters, except for cortical bone density. In addition, the same pathway is observed through the correlation between obesity and bone parameters. Thus, in the obese population, assessing cortical density might help the physician to identify bone alteration. As we included only healthy young older adults living in the community and performed a secondary analysis (not the main outcome), these results need to be confirmed using RCT to refine our findings and inflammaging status need to be explored as potential confounders.

### Supplementary Information

Below is the link to the electronic supplementary material.

Supplementary file1 (DOCX 27 KB)

## Data Availability

Data are available via corresponding according to country agreements and on reasonable request.
