# Supplementary material for: Relationships between obesity markers and bone parameters in community-dwelling older adults
Source: Aging Clin Exp Res. 2024 Feb 29;36(1):49. doi: 10.1007/s40520-023-02673-8 (PMC10904426; doi:10.1007/s40520-023-02673-8)
Supplement: Supplementary file 1 — Supplementary file1 (DOCX 27 KB) [file 40520_2023_2673_MOESM1_ESM.docx]

**Table S1. Comparison of obesity and bone parameters between obese and non-obese men according to obesity criteria**

| Variable | **BMI** | | **WC** | | **FM** | |
| --- | --- | --- | --- | --- | --- | --- |
|  | Obese (n=29) | Non obese (n=85) | Obese (n=93) | Non obese (n=21) | Obese (n=77) | Non obese (n=37) |
| **ANTHROPOMETRIC DATA** | | | | | | |
| **BMI (kg/m^2^)** | 33.2±2.6 | 26.3±2.0** | 29.0±3.4 | 23.6±2.7** | 29.5±3.5 | 25.0±2.7** |
| **WC (cm)** | 113±12 | 100±9** | 107±8 | 85±8** | 107±10 | 95±10** |
| **BODY COMPOSITION (DXA)** | | | | | | |
| **Total FM (%)** | 37.5±4.5 | 28.4±6.0** | 32.6±5.3 | 22.4±7.0** | 34.4±4.2 | 23.0±5.0** |
| **Total BMD (g/cm^2^)** | 1.25±0.10 | 1.24±0.10 | 1.25±0.10 | 1.19±0.10* | 1.24±0.10 | 1.24±0.12 |
| **Spine BMD (g/cm^2^)** | 1.22±0.12 | 1.26±0.12 | 1.27±0.20 | 1.18±0.20* | 1.24±0.17 | 1.27±0.20 |
| **Hip BMD (g/cm^2^)** | 1.07±0.15 | 1.05±0.13 | 1.06±0.10 | 1.04±0.14 | 1.06±0.13 | 1.05±0.13 |
| **BONE VARIABLES (pQCT)** | | | | | | |
| **Cortical bone density (mg/cm³)** | 1095±26 | 1102±33 | 1097±32 | 1114±27* | 1096±31 | 1110±31* |
| **Cortical bone area (mm²)** | 447±46 | 418±49** | 429±45 | 405±62* | 429±48 | 415±51 |
| **Total bone density (mg/cm³)** | 723±83 | 711±89 | 717±84 | 697±102 | 719±78 | 703±104 |
| **Total bone area (mm²)** | 715±78 | 681±91 | 690±90 | 683±88 | 686±82 | 692±104 |
| **SSI (mm³)** | 3564±544 | 3347±509* | 3402±504 | 3382±615 | 3386±530 | 3422±516 |
| **IPo (mm^4^)** | 70275±14070 | 63902±13867* | 65962±13906 | 63011±15097 | 65875±13499 | 64453±15443 |
| **METABOLIC STATUS** | | | | | | |
| **Metabolic syndrome** | 11(38%) | 33 (38%) | 42 (57%) | 2 (10%)** | 30 (39%) | 14 (38%) |

**Table S2. Comparison of obesity and bone parameters between obese and non-obese women according to obesity criteria**

| Variable | **BMI** | | **FM** | |
| --- | --- | --- | --- | --- |
|  | Obese (n=19) | Non obese (n=35) | Obese (n=23) | Non obese (n=31) |
| **ANTHROPOMETRIC DATA** | | | | |
| **BMI (kg/m^2^)** | 35.3±5.0 | 25.5±2.7** | 32.2±5.7 | 24.6±2.6** |
| **WC (cm)** | 113±10 | 93±9** | 107±12 | 89±7** |
| **BODY COMPOSITION (DXA)** | | | | |
| **Total FM (%)** | 48.2±5.6 | 38.5±5.0** | 46.3±5.2 | 36.0±4.2** |
| **Total BMD (g/cm^2^)** | 1.20±0.10 | 1.10±0.10** | 1.17±0.10 | 1.09±0.10** |
| **Spine BMD (g/cm^2^)** | 1.18±0.20 | 1.05±0.20* | 1.12±0.20 | 1.06±0.20 |
| **Hip BMD (g/cm^2^)** | 1.02±0.16 | 0.91±0.12* | 0.98±0.15 | 0.90±0.14* |
| **BONE VARIABLES (pQCT)** | | | | |
| **Cortical bone density (mg/cm³)** | 1075±40 | 1090±29 | 1083±33 | 1087±36 |
| **Cortical bone area (mm²)** | 353±44 | 336±35 | 348±37 | 334±40 |
| **Total bone density (mg/cm³)** | 696±132 | 711±81 | 705±116 | 706±77 |
| **Total bone area (mm²)** | 598±101 | 537±61* | 577±92 | 534±57* |
| **SSI (mm³)** | 2507±387 | 2290±333* | 2442±354 | 2265±347* |
| **IPo (mm^4^)** | 47499±9034 | 40644±7077** | 45061±8549 | 40354±7584* |
| **METABOLIC STATUS** | | | | |
| **Metabolic syndrome** | 10 (53%) | 11 (31%) | 15 (65%) | 6 (19%) |
